# Chemotherapy-Induced Cardiotoxicity: Overview of the Roles of Oxidative Stress

**DOI:** 10.1155/2015/795602

**Published:** 2015-09-29

**Authors:** Paweorn Angsutararux, Sudjit Luanpitpong, Surapol Issaragrisil

**Affiliations:** Siriraj Center of Excellence for Stem Cell Research, Faculty of Medicine Siriraj Hospital, Mahidol University, Bangkok 10700, Thailand

## Abstract

Chemotherapy-induced cardiotoxicity is a serious complication that poses a serious threat to life and limits the clinical use of various chemotherapeutic agents, particularly the anthracyclines. Understanding molecular mechanisms of chemotherapy-induced cardiotoxicity is a key to effective preventive strategies and improved chemotherapy regimen. Although no reliable and effective preventive treatment has become available, numerous evidence demonstrates that chemotherapy-induced cardiotoxicity involves the generation of reactive oxygen species (ROS). This review provides an overview of the roles of oxidative stress in chemotherapy-induced cardiotoxicity using doxorubicin, which is one of the most effective chemotherapeutic agents against a wide range of cancers, as an example. Current understanding in the molecular mechanisms of ROS-mediated cardiotoxicity will be explored and discussed, with emphasis on cardiomyocyte apoptosis leading to cardiomyopathy. The review will conclude with perspectives on model development needed to facilitate further progress and understanding on chemotherapy-induced cardiotoxicity.

## 1. Introduction

Chemotherapy is a common treatment for various cancers as an adjuvant or a primary therapy; however, it also carries a risk of adverse effects that might leave an unfavorable damage to cancer patients. Chemotherapy-induced cardiotoxicity is a serious complication that limits the clinical use of chemotherapeutic agents, particularly the anthracyclines, since it could eventually culminate in the development of life-threatening cardiomyopathy. Understanding the mechanisms that are responsible for cardiotoxicity could help lessen the undesirable impact on normal tissues and improve the cancer treatment regimen. One of the most widely accepted mechanisms of chemotherapy-induced cardiotoxicity involves the generation of oxidative stress. This review provides an overview of the roles of oxidative stress in chemotherapy-induced cardiotoxicity. Current understanding in the molecular mechanisms of oxidative stress-mediated cardiotoxicity is discussed, with emphasis on cellular apoptosis leading to cardiomyopathy.

## 2. Chemotherapy-Induced Cardiotoxicity

The basic principle of chemotherapy is to impair mitotic and metabolic process of cancer cells. Unfortunately, certain normal cells and tissues are also affected by the chemotherapy, leading to various mild and severe adverse effects, including nausea and vomiting, bone marrow suppression, and cardiovascular side effects, namely, hypotension, tachycardia, arrhythmias, and heart failure [1]. The National Cancer Institute generally defines toxicity that affects the heart as cardiotoxicity. Although cardiotoxicity could be affected by different chemotherapeutic agents as summarized in Table 1, certain class of chemotherapy, for example, the anthracyclines, induces more common and frequent adverse effects. These drugs should not be administered to patients with high risk of developing cardiac complications. The risk of cardiotoxicity increases in patients with hypertension, diabetes mellitus, liver disease, and previous cardiac diseases [2]. The risk of cardiotoxicity also depends largely on route of administration, duration of chemotherapy (cumulative dose) and the dosage regimen. For example, a combination of anthracyclines with other potential cardiotoxic agents, namely paclitaxel or trastuzumab, would greatly enhance the risk of cardiotoxicity that potentially lead to disastrous congestive heart failure [3–6].

Anthracyclines including doxorubicin (DOX), daunorubicin, epirubicin, and idarubicin are highly effective against acute lymphoblastic and myeloblastic leukemias. DOX in particular has been found to have a much broader spectrum, which includes numerous solid tumors, for example, breast cancer, sarcomas, and childhood solid tumors (e.g., Wilms' tumor) in addition to hematological malignancies, for example, leukemias, Hodgkin's disease, and non-Hodgkin's lymphomas [1]. Anthracycline-induced cardiotoxicity can occur in an acute or chronic manner. The acute cardiotoxicity within the course of treatment or immediately afterwards including pericarditis-myocarditis or arrhythmias is normally reversible and generally manageable, while the chronic cardiotoxicity, which may manifest even decades after the completion of treatment, is serious and clinically significant, substantially affecting overall morbidity and mortality and requiring long-term therapy. As DOX is an important component of current cancer treatment that generates high level of ROS, this review mainly emphasizes on the DOX-induced cardiotoxicity.

### 2.1. DOX-Induced Cardiotoxicity

The cumulative dose of administered DOX is an important factor that dictates its cardiotoxicity; it cannot exceed 500 mg/m^2^ or else the risk of congestive heart failure (CHF) would increase tremendously [7]. The chance of developing CHF increases from <4% at the cumulative dose of 500–550 mg/m^2^ to <18% or 36% when the cumulative dose of DOX is 551–600 or over 600 mg/m^2^, respectively. A retrospective analysis revealed that 30-year childhood cancer survivors had a 15-fold higher rate of heart failure and 10-fold higher rate of other cardiovascular diseases when compared to estimated values in their siblings. Cardiac abnormality can be seen as asymptomatic disturbances in cardiac rhythms [8], changes in blood pressure [9], thickening of pericardium [10], cardiac dilation [11], and cardiomyopathy.

Histologically, areas of patchy and interstitial fibrosis with scattered vacuolated cardiomyocytes can be visualized under microscope [11, 12]. There are also partial loss of myofibrils and the degeneration of myocyte vacuoles [13], resulting in the remaining peripheral Z disks without filaments and the large membrane-bound space accordingly. In the nucleus, chromatin disorganization with partial replacement of the chromatin by pale staining fibers and filaments could be observed [11]. Ultimately, these changes would lead to cardiomyocyte death. Since the turnover rate of cardiomyocytes in the heart is very low, approximately 1% at the age of 20 and lower in older ages [14], the progressive loss of myocytes cannot be reversed. Subsequently, the heart loses its ability to function properly. These pathophysiological changes can lead to dilated cardiomyopathy, cardiac hypertrophy, and eventually heart failure.

DOX is particularly harmful to the heart due to its exceptional effects on mitochondria. Heart as a pump that circulates blood throughout the body requires a great amount of energy and thus contains a large amount of energy producing mitochondria. Generally, mitochondria are the site where most of reactive oxygen species (ROS) are produced as a consequence of electrons escaping from electron transport chain and captured by oxygen, rendering it to be the home of superoxide production. However, DOX can drive these ROS production to another level. Enzymes within mitochondria, including NADPH oxidase (also known as NADPH dehydrogenase), cytochrome P-450 reductase, and xanthine oxidase (XO), can transform DOX and other anthracyclines in the form of quinone to semiquinone via one electron reduction of the quinone moiety in ring C [14–17]. This semiquinone can be readily regenerated back to its parental quinone by reacting with oxygen, generating superoxide anion (O_2_^∙−^), which could be further changed to other ROS or reactive nitrogen species (RNS) [18, 19]. This redox cycling thus aids in amplifying the production of oxidative stress. Apart from mitochondrial enzymes, endothelial nitric oxide synthase (eNOS), which generates nitric oxide (NO), can also affect the ROS production by DOX [20]. Expression and transcription of eNOS were found to be increased after the DOX administration [19, 21]. By binding to eNOS's reductase domain, DOX veers its production towards more O_2_^∙−^ and less nitric oxide (NO).

The nature of cardiac tissue that exhibits low level of antioxidant enzymes such as superoxide dismutase (SOD) and catalase makes it more susceptible to ROS generation and accumulation of oxidative stress [22]. Moreover, DOX is known to bind to cardiolipin, a major phospholipid component of heart mitochondrial inner membranes, with high affinity [23–25]. DOX and its major metabolite doxorubicinol (DOXol) are abundantly retained inside cardiac cells, attributable to cardiotoxicity [26, 27].

## 3. Oxidative Stress

Major mechanism of chemotherapy-induced cardiotoxicity involves the generation of ROS. Elevated ROS that causes cellular damages and alteration responses, referred to as oxidative stress, occurs when the delicate balance of the ROS-generating system and antioxidant defense system was tipped in favor of the former. Immediate systemic oxidative stress and reduced antioxidant status, as demonstrated by the decrease in glutathione (GSH) and total antioxidant capacity of plasma (TRAP) levels, were observed in cancer patients receiving DOX treatment [28]. To discuss the advances in molecular mechanisms underlying chemotherapy-induced cardiotoxicity, an overview of biochemistry of ROS and antioxidants has provided.

Basically, ROS are reactive molecules that contain oxygen atom and are primarily generated either by metabolic process or by physical irradiation as O_2_^∙−^. The reaction is catalyzed by NADPH oxidase with electron supplied by NADPH as in the following equations:

(1) O2+e−⟶O2∙−2O2+NADPH⟶2O2∙−+NADP++H+

In mitochondrial electron transport chain, O_2_^∙−^ is also generated when an electron is transferred from NADH to oxygen molecule by NADH-ubiquinone oxidoreductase or complex I:

(2) $$\begin{array}{c} NADH+H^{+}+2O_{2}⟶{NAD}^{+}+2H^{+}+{O_{2}}^{∙-} \end{array}$$

O_2_^∙−^ then undergoes a dismutation, a reaction that is accelerated by SOD enzyme, generating less-reactive specie hydrogen peroxide (H_2_O_2_):

(3) $$\begin{array}{c} 2{O_{2}}^{∙-}+2H\overset{SOD}{⟶}H_{2}O_{2}+O_{2} \end{array}$$

The H_2_O_2_-removing enzymes include catalase and glutathione peroxidase (GPx):

(4) H2O2→Catalase2H2O+O22GSH+H2O2⟶GPxGSSG+2H2O

O_2_^∙−^ may react with H_2_O_2_ and form the highly reactive hydroxyl radical (^∙^OH) by Haber-Weiss reaction:

(5) $$\begin{array}{c} {O_{2}}^{∙-}+H_{2}O_{2}⟶{OH}^{-}+{OH}^{∙}+O_{2} \end{array}$$

Since the Haber-Weiss reaction is kinetically slow, transition metals such as iron and copper often serve as its catalysts. The iron-catalyzed Haber-Weiss reaction is called Fenton reaction:

(6) $$\begin{array}{c} {Fe}^{2+}+H_{2}O_{2}⟶{Fe}^{3+}+{OH}^{-}+{OH}^{∙} \end{array}$$

Under stress conditions, O_2_^∙−^ facilitates the release of free iron from iron-containing molecules, for example, [4Fe-4S]-containing enzymes, leading to the generation of ^∙^OH from Fenton reaction. Moreover, O_2_^∙−^ can facilitate the ^∙^OH production in the reaction by generating ferrous (Fe(II)) from the reduction of ferric (Fe(III)):

(7) $$\begin{array}{c} {Fe}^{3+}+{O_{2}}^{∙-}⟶{Fe}^{2+}+O_{2} \end{array}$$

With regard to DOX, iron has been implicated in its redox cycling [29, 30]. When DOX was reduced to semiquinone radical, it can form a complex of semiquinone and iron-free radical, which would spontaneously revert back to DOX while generating O_2_^∙−^ [31], and thus it magnifies the generation of DOX-derived ROS. However, under normal situation, iron is sequestered within the cells by ferritin as polynuclear ferric oxyhydroxide [32]. DOX's metabolite DOXol was shown to remove iron from Fe-S cluster of cytoplasmic aconitase, an enzyme in the Krebs cycle, and turn it into cluster-free iron regulatory proteins-1 (IRP-1) [33]. Then, via redox reaction, DOX was regenerated and formed complex with Fe(II), blocking the conversion of IRP-1 back to aconitase [34]. This IRP-1 then bound to iron-responsive elements (IREs) in ferritin mRNA, inhibiting its translation and resulting in the tremendous release of free iron within the cells [35, 36].

In addition to its direct effects on DNA, RNA, proteins, and lipids, ROS can also act as secondary signaling molecules in various pathways that are involved in homeostasis, including cell proliferation and cell death [37, 38]. Thus, it is imperative to maintain the proper level of ROS in the cellular environment. In the heart, oxidative stress could lead to cellular hypertrophy [39], gene expression alterations [40], cell death activation [41], extracellular matrix (ECM) transformation [42], ventricular remodeling [41], and calcium transient perturbation [43], all of which could result in the pathophysiological changes that lead to cardiomyopathy and heart failure.

## 4. Experimental Models

Similar to other abnormalities, the ideal and most relevant data for human cardiotoxicity should be obtained from suffering patients. However, due to the limitation in obtaining human myocardial biopsy or necropsy and the complication from patients' multidrug regimen, the meaningful information for chemotherapy-induced cardiotoxicity comes mostly from experimental models. The commonly used animals for* in vivo* studies include mouse, rat, rabbit, pig, and dog. These whole animal studies enable repeated administration of chemotherapeutic agents, mimicking chronic cardiotoxicity in clinical practice. In addition, transgenic animal models with certain gene modification could aid tremendously to study and model cardiotoxicity. For example, Hfe-deficient mice with excess iron stores exhibited increased susceptibility to DOX-induced cardiotoxicity, suggesting the significance of iron in cardiotoxicity [44]. On the other hand, the simplest and most straightforward approach is the use of* in vitro* cell culture models, including isolated cardiac myocytes, particularly primary neonatal rat cardiomyocytes and less often adult cardiomyocytes, and cardiomyocyte-derived cell lines, for example, H9c2 rat embryonic cardiomyoblasts [45]. These* in vitro* models offer the advantages of gene manipulation that provides insight into the molecular mechanisms, high throughput, ease of use, and low cost.

## 5. Molecular Mechanisms of Chemotherapy-Induced Cardiotoxicity

Extensive researches have been conducted to examine the molecular mechanisms of cardiotoxicity induced by chemotherapy, particularly DOX, and oxidative stress was found to be the major contributor to various potential causes that ultimately lead to cardiomyopathy and heart failure. We will summarize the current understanding of ROS in such phenomena and discuss the alterations of key related genes and/or proteins, with emphasis on cellular apoptosis leading to cardiomyopathy.

### 5.1. Cellular Hypertrophy

Cellular hypertrophy is characterized by an increase in cell size and volume, enhanced protein synthesis, and content and heightened organization of the sarcomere [46, 47]. At the molecular level, there is an induction of cardiac hypertrophic-associated genes such as ventricular myosin light chain-2 (MLC-2v), alpha-myosin heavy chain (*α*-MHC), and atrial natriuretic peptide (ANP) [48]. Major signaling cascades of cardiac hypertrophy include tyrosine kinases (Src and focal adhesion kinase), protein kinase C (PKC), mitogen activated protein kinases (MAPK; ERK1/2, p38, and JNK), calcineurin, PI3K/Akt, and NF-*κ*B, coupling to the activation of transcriptional expression of hypertrophic-associated genes. It is now evident that ROS are capable of direct modulation of these cascades [39, 41, 49–52]. Additionally, ROS were found to activate apoptosis signal-regulating kinase 1 (ASK-1), which in turn stimulates p38 and JNK MAPK as well as NF-*κ*B pathways [53–55]. Merten et al. reported that DOX-induced H9c2 cardiac hypertrophy involved the activation of PI3K/Akt and to a lesser extent calcineurin [56]. The role of oxidative stress in DOX-induced cardiac hypertrophy was supported by the attenuation of DOX-induced hypertrophy and cardiotoxicity in transgenic mice containing high level of cardiac metallothionein, a potent antioxidant [57].

### 5.2. ECM Remodeling

Microenvironment is crucial to the proper functions of cardiomyocytes. ECM microenvironment provides a platform for cardiomyocytes to attach, align, and orient, thereby facilitating efficient cellular contraction, proper force transduction, and smooth electrical transmission [58]. An alteration in ECM structure or composition would transform the healthy functional heart to the defected one [59–61]. Matrix metalloproteinases (MMPs) are enzymes that are responsible for the ECM degradation. It has previously been demonstrated that ROS activated MMPs and ECM degradation via the activation of activator protein-1 (AP-1) and NF-*κ*B, leading to the impairment of cardiac functions [62, 63]. Animal studies in mouse and rat showed that MMPs particularly MMP-2 and MMP-9 mediated DOX-induced cardiotoxicity [64–66]. DOX increased MMP-2 and MMP-9 activities through the mechanisms that involved the activation of p38 MAPK and NADPH oxidase, respectively [67]. Further, Nox2 NADPH oxidase was found to be a major source of DOX-derived ROS. Cardiac remodeling induced by DOX was less pronounced in Nox2-deficient mice (Nox2^−/−^) compared to the wild-type controls [68]. Additionally, DOX induced nitric oxide (NO)* in vitro* and* in vivo* [69, 70], which in the presence of O_2_^∙−^ formed peroxynitrite (ONOO^−^), leading to an activation of precursors of MMPs (pro-MMPs) and ECM remodeling.

### 5.3. Impaired Cardiac Contraction

Cardiomyocytes (also known as cardiac muscle cells) are myogenic, having an inherent rhythmic contraction and relaxation. For the whole heart to properly pump and propel blood into the circulation, precise control and synchronization of these cells are imperative. Each cardiomyocyte is composed of bundles of myofibrils, which is a chain of several contractile units called sarcomeres. The sarcomeres contain thick (myosin) and thin (actin) myofilaments that process cardiac contraction. In the relaxed state, tropomyosin wrapped around actin filament and covered the myosin-binding site. Calcium ion, generally resides within sarcomeric reticulum, is a key player for myocyte contraction. Once released to cytosol, calcium ions bind to troponin, which is associated with tropomyosin, and shift tropomyosin position on actin filament, exposing the myosin-binding site for myosin head of thick filament to bind to the thin filament and resulting in sarcomere shortening. That being said, what control cardiac contraction are the myofilaments and calcium flux.

DOX could affect the transcription and expression of cardiac specific proteins [11, 71, 72]. GATA4 is a transcription factor critical for regulation of cardiac differentiation, sarcomere synthesis, and cell survival. GATA4 is expressed in the cardiomyocytes where it functions to regulate many cardiospecific genes, including cardiac adriamycin-responsive protein (CARP). DOX-derived ROS downregulated GATA4 binding activity, leading to myofibrillar deterioration, disruption of sarcomere organization, and reduction of contractile function [73, 74].

DOX has been shown to alter calcium homeostasis [75, 76]. This may be, in part, due to the change of ion channel flux and the functional defect of membrane ion pump [77] as a result of lipid peroxidation by DOX-derived ROS. ^∙^OH and H_2_O_2_ can readily attack polyunsaturated fatty acids of the membrane and initiate a redox chain reaction that destroys membrane lipids [78], thereby disturbing the function of membrane-bound protein including mitochondrial calcium channel [17]. Moreover, DOX could directly affect the expression level of important component of sarcomeric reticulum calcium release or uptake channels, for example, ryanodine receptor (RYR2) and sarco-/endoplasmic reticulum Ca^2+^ ATPase (SERCA2) [79, 80], leading to an impaired calcium handling and subsequent impaired cardiac contraction [81]. It has been reported that DOX modifies the thiol group (–SH) of RYR2 [82, 83]. This modification enhances the probability of RYR2 channel to adopt its open state [84, 85], making cytosol overloaded with calcium ions. Additionally, DOX could increase the activity of voltage-sensitive L-type calcium channel (VSCC) on cardiac cell membrane [86] as well as inhibiting the Na^+^/Ca^2+^ exchanger (NCX) on sarcolemmal membrane [87], resulting in calcium overload.

When cytosolic calcium ions were excessive, the relaxed state after contraction would be undermined, and thus the contraction-relaxation cycle of cardiomyocytes is interrupted. DOX's major metabolite DOXol was shown to have more profound effect on contraction-relaxation cycle as compared to DOX [26]. DOXol could inhibit RYR2, Na^+^/K^+^ pump on the cell membrane, and proton pump on mitochondria, resulting in the impairment of relaxation [26, 88].

### 5.4. Cardiac Cell Death

**Figure 1:**
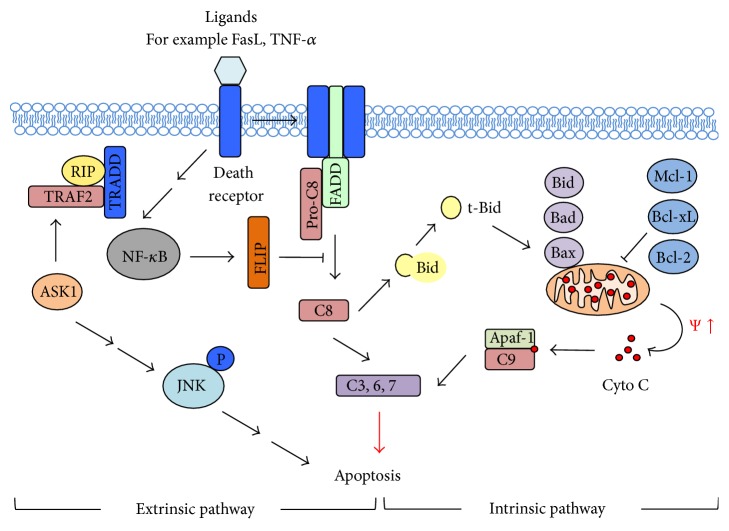
Major pathways of apoptosis. An extrinsic pathway of apoptosis (*left*) involves the stimulation through binding of death receptor (e.g., FasR and TNFR) to their respective ligands (e.g., FasL and TNF-*α*), recruiting adaptive proteins such as Fas-associated death-domain (FADD) and pro-caspase-8 (pro-C8), forming death-inducing signaling complex (DISC) and relaying signals to activation of effector caspases such as caspase-3 (C3), C6, and C7. In addition, Bid is also activated, which transduces these death signals to the intrinsic pathway. On the other hand, an intrinsic pathway of apoptosis is induced in response to cellular stresses such as DNA damage and ROS that increase the expression of proapoptotic Bcl-2 family proteins (e.g., Bid, Bad, and Bax), while repressing antiapoptotic Bcl-2 family proteins (e.g., Bcl-2, Bcl-xL, and Mcl-1), leading to an alteration in mitochondrial membrane potential and the release of cytochrome C (Cyto C). Proteins such as Apaf-1 and caspase-9 (C9) are activated, resulting in the formation of an apoptosome, which then stimulate the activation of effector caspases. NF-*κ*B and JNK/ASK-1 also play a role in apoptotic signaling through the regulation of antiapoptotic molecules such as FLIP and Bcl-2.

Programmed cell death or apoptosis is a critical cellular process occurring when a cell commits suicide, as it is no longer needed, no longer processes properly or senses the cytotoxic signals. Damage to genetic DNA, proteins, cellular organelles, or membrane integrity that is beyond repair would trigger apoptosis in order to reserve resources and energy for other normal cells and prevent neighboring cells from its death signals. Apoptosis is a tightly regulated process, involving multiple signaling cascades commonly in extrinsic death receptor and intrinsic mitochondrial apoptosis pathways (Figure 1).

The death receptor pathway involves the binding of death ligands, for example, Fas ligand (FasL) and TNF-*α*, to their respective membrane-bound death receptors, for example, Fas receptor or apoptosis antigen-1 (APO-1) or CD95 and TNF-*α* receptor (TNFR), resulting in the recruitment of secondary proteins that will relay the signals to various proteins mediating the caspase cascade, leading to apoptosis. In particular, when FasL binds to Fas receptor, the receptor oligomerizes and recruits an adaptor protein Fas-associated death-domain (FADD) and pro-caspase-8, assembling the death-inducing signaling complex (DISC) and activating the initiator caspase-8 (FLICE), which in turn activates effector caspase-3 [89]. Caspase-3 catalyzes the cleavage of poly(ADP-ribose) polymerase (PARP), causing apoptosis as characterized by chromatin condensation, DNA fragmentation, and membrane blebbing [90]. Alternatively, caspase-8 can cleave Bid to become truncated Bid (t-Bid) that will translocate to mitochondria, thus linking the death receptor and mitochondrial pathways [91, 92]. Similar to FasL, the binding of TNF-*α* to TNFR recruits TNFR-associated death domain protein (TRADD) adaptor protein, which will then recruit either FADD or receptor-interacting protein (RIP). These two pathways result in two different situations that are TRADD/FADD activates caspase-8 and induces apoptosis, while TRADD/RIP/TRAF2 activates NF-*κ*B and prosurvival pathway [93]. NF-*κ*B could induce the expression of antiapoptotic proteins such as FLIP and Bcl-2 [94]. On the other hand, TRAF-2 can activate ASK-1/JNK pathway via an ROS-dependent manner and induce apoptotic signaling cascades [95]. An active phosphorylated JNK can translocate to nucleus and increase the expression of proapoptotic genes. Or else, it can translocate to mitochondria and phosphorylate Bcl-2 to antagonize its activity as well as releasing the cytochrome C and Smac/Diablo (Smac), which is a TRAF2/IAP1 inhibitor.

The mitochondrial pathway relies on the changes in mitochondrial transmembrane potential (*ψ*), which is a key indicator of mitochondrial membrane permeability. Bcl-2 family proteins includes antiapoptotic proteins, such as Bcl-2, Bcl-XL, and Mcl-1, and proapoptotic proteins, such as Bid, Bad, Bax, Bak, Bok, and Bim. The proapoptotic proteins facilitate the release of cytochrome C from mitochondria by dimerizing and forming transition pores [96]. The released cytochrome C subsequently binds to caspase adaptor molecule Apaf-1 and recruits initiator pro-caspase-9 to form a complex called apoptosome, which activates effector caspase-3. The oligomerization of proapoptotic Bcl-2 family proteins can be prevented by antiapoptotic Bcl-2 family proteins.

When the number of cardiomyocytes available to function in the heart was remarkably reduced, the heart can no longer effectively and efficiently pump blood, sequentially leading to ventricular remodeling and heart failure [97–100]. DOX has been demonstrated to cause cardiomyocyte apoptosis by various means. It can activate mitochondrial apoptosis pathway by disrupting the cardiolipin, an important component of mitochondrial inner membrane [99]. Cardiolipin controls energy metabolism, and thereby its alteration can potentially lead to heart failure [101]. Apoptosis can also be activated by the damage to cellular lipid membrane, DNA, or mitochondrial DNA induced by DOX [102–105]. ROS are believed to be responsible for the apoptotic effect of DOX. An increase in ROS level as measured by electron spin resonance spectroscopy was observed in cardiac cells after exposing to DOX, in agreement with an increase in malondialdehyde, a product of lipid peroxidation driven by ROS [106, 107]. Mitochondrial DNA is particularly vulnerable to DOX toxicity due to its proximity to DOX and sources of ROS generation within mitochondrial membrane [108]. In addition to the amplification of ROS signals, as mentioned earlier, DOX suppressed the antioxidant defense system. DOX reduced the levels and activities of antioxidant GSH, GPx-1, and sulfhydryl groups within enzyme complex as well as the cytosolic SOD, making cells vulnerable to oxidative stress [22, 31, 49, 108–113]. A large amount of O_2_^∙−^ produced within mitochondria cannot pass through its membrane and thus can easily damage mitochondrial DNA that does not have the protective complex chromatin organization like histone proteins and introns and has limited repair capability [114].

**Figure 2:**
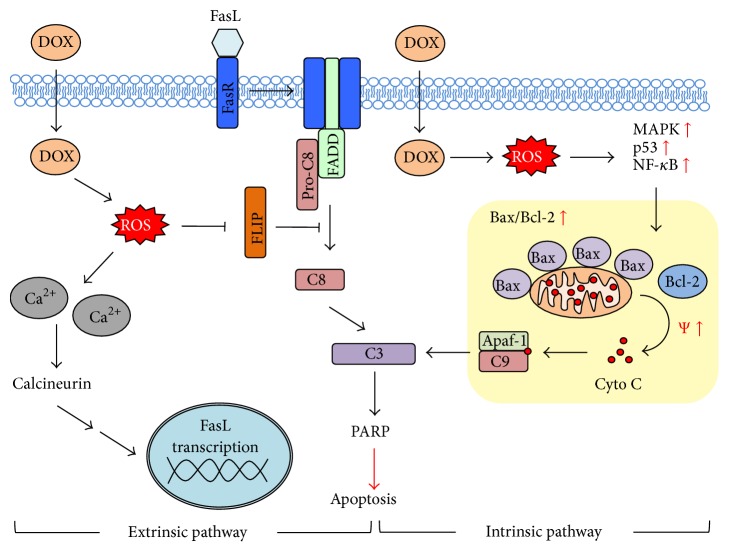
Schematic representation of DOX-induced apoptosis and the involvement of ROS. DOX-derived ROS could affect the regulation of calcium homeostasis, resulting in cytosolic calcium (Ca^2+^) overload that can activate calcineurin and increase the transcription of Fas ligand (FasL). DOX-derived ROS could also inhibit the expression of caspase-8 (C8, also known as FLICE) inhibitory protein FLIP, rendering cells to apoptosis. Additionally, DOX-derived ROS could act as an intrinsic stress that activates mitogen activated protein kinases (MAPK) p38 and JNK and NF-*κ*B pathways as well as intracellular p53 accumulation, leading to an alteration in the ratio of proapoptotic proteins to antiapoptotic proteins (e.g., Bax to Bcl-2), cytochrome C (Cyto C) release, and caspase-9 and -3 (C9/C3) activation.

With regard to apoptosis pathways, DOX-derived ROS was reported to activate p53 as well as p38 and JNK MAPK and NF-*κ*B signaling pathways, resulting in an imbalance between pro- and anti-apoptotic Bcl-2 family proteins, for example, an increase in Bax to Bcl-2 ratio [115, 116] (Figure 2). Such imbalance disrupted mitochondrial membrane potential, causing cytochrome C release. Notably, JNK can also translocate to nucleus after being activated by DOX-derived ROS and phosphorylate c-Jun, that subsequently binds to AP-1 and initiates FasL transcription [95]. Inhibition of FLIP by DOX-derived ROS further rendered cardiac cells to apoptosis induced by FasL, providing another potential mechanism for DOX-mediated apoptosis [117]. Additionally, DOX-derived ROS were shown to cause calcium leakage from sarcoplasmic reticulum, which then activated calcineurin and promoted apoptosis via NFAT/FasL activation or Bcl-XL and Akt pathway inhibition by Bad [118]. Several studies reported that DOX induced the loss of phospholipase C delta-1 (PLCD-1) and PLCD-3 that simultaneously led to cardiomyocyte apoptosis and the development of cardiomyopathy [118, 119]. Additionally, DOX could directly induce the mitochondrial release of cytochrome C and activate caspase-3 or p53 accumulation, leading to the proapoptotic signaling cascades [120, 121].

### 5.5. Autophagy

The role of autophagy towards cell fate may vary, depending on cellular environment and stimuli. Under normal physiological condition, autophagy enhances cellular function and overall viability by eliminating damaged and/or unnecessary proteins and organelles, while maintaining cellular integrity and inhibiting apoptosis. Under pathological condition, autophagy could either promote cell survival or induce cell death. Several studies demonstrated cardiac autophagy upon DOX administration, as evaluated by an increase of autophagic vacuoles and expression of autophagosome markers [122–127]; however, its precise role in DOX toxicity still remains controversial. Many groups agreed that inhibition of autophagy resulted in a decrease in DOX toxicity [122, 125, 127, 128], in correspondence with a decrease in mitochondrial membrane potential and ROS generation [127]. On the other hand, Kawaguchi et al. and Sishi et al. observed the protective effect of autophagy towards DOX cardiotoxicity [124, 129], likely through the reduction in mitochondrial ROS [129]. 

## 6. Preventive Strategies

Cardiotoxicity could be life threatening, and thus several attempts have been made to attenuate and minimize such toxicity induced by chemotherapy, particularly the most common DOX. Clinical practice suggests close monitoring and evaluation of patient risks for developing complications after treatment. Early detection and immediate proper medication could reverse the condition in time that minimizes cardiotoxic effects. Analogues of DOX with some changes in molecular structures, including epirubicin, idarubicin, and mitoxantrone, have been developed and became another appealing alternative as studies in cancer patients showed comparable drug efficacy with lower cardiotoxicity to conventional DOX [130–132]. Liposomal DOX is another strategy to reduce the drug toxicity as encapsulating DOX was restricted to the site with tight capillary junction like in the heart's wall, while readily penetrating through the more fragile tumor vasculature [133].

Dexrazoxane is the only FDA-approved cardioprotective agent against cardiotoxicity induced by anthracyclines. Due to the potential risk of developing secondary tumors and interfering effect of dexrazoxane towards anticancer activity of DOX, Dexrazoxane clinical use is limited only to some certain groups of patients [134, 135], namely, adult patients with breast cancer who have received cumulative dose of at least 300 mg/m^2^ doxorubicin or 540 mg/m^2^ epirubicin.

## 7. Conclusions and Perspectives

Adverse effects of chemotherapeutic agents limit their clinical benefits. For instance, DOX, one of the most effective and frequently used chemotherapeutic agents to treat a variety of cancers, poses serious problem of cardiotoxicity when administered over certain critical dosage. Several studies demonstrate that ROS are the main contributor of DOX-induced cardiotoxicity. DOX-derived ROS affected various major cellular processes leading to cardiomyopathy and eventually congestive heart failure, including cellular hypertrophy, ECM remodeling, impaired cardiac contraction, and remarkable cardiac cell death. Specifically to cell death, DOX-derived ROS served as a secondary signaling molecule engaged in numerous pathways of apoptosis. The protective effect of antioxidants against DOX-induced apoptosis in cell culture and animal models support the notion that DOX-induced cardiotoxicity is caused mainly by ROS. Other chemotherapeutic agents that induce ROS and high degree of cardiotoxicity include all other anthracyclines, alkylating agents, and vinca alkaloids. However, the nonprotective effect of antioxidants against cardiotoxicity induced by DOX and other chemotherapeutic agents was observed, particularly in chronic studies using animal models or in clinical trials. Such failure of antioxidants may be due to (i) a complexity of ROS network that partly interruptions or inhibitions by antioxidants cannot totally reverse its effects and (ii) a variety of different mechanisms governing in chemotherapy-induced cardiotoxicity. A key to effective preventive strategies is a better understanding of molecular mechanisms of chemotherapy-induced cardiotoxicity which requires the development of more predictive experimental models. Much of our understanding on chemotherapy-induced cardiotoxicity was based on animal models and neonatal rat cardiomyocyte culture, thus strengthening the notion that the experimental models that mimic human heart physiology and allow mechanistic studies are currently lacking. The newly developed human induced pluripotent stem- (iPS-) derived cardiomyocytes or three-dimensional (3D) organ culture is promising and might provide us with the different facet of this complicated problem. In addition, the drug concentrations and regimen used during each preventive study should faithfully reflect the real scenario of drug administration during cancer therapy so that its results could be better appreciated and reliable.

## Figures and Tables

**Table 1:** Potential cardiotoxicity induced by numerous chemotherapeutic agents.

Class	Examples of chemotherapeutic agents	Possible cardiotoxicity
Anthracyclines and anthraquinones	DOX, mitoxantrone	Congestive heart failure, left ventricular dysfunction, acute myocarditis, and arrhythmia

Antimetabolite agents	Capecitabine, 5-fluorouracil, and cytarabine	Ischemia, pericarditis, congestive heart failure, and cardiogenic shock

Antimicrotubule agents	Paclitaxel, vinca alkaloids	Sinus bradycardia, ventricular tachycardia, atrioventricular block, hypotension, congestive heart failure, and ischemia

Alkylating agents	Cyclophosphamide, ifosfamide	Neurohumoral activation, mild mitral regurgitation

Monoclonal antibody against HER2	Trastuzumab	Arrhythmias, congestive heart failure, angioedema, and left ventricular dysfunction

Antiangiogenic and tyrosine kinase inhibitors	Imatinib, sorafenib, and sunitinib	Hypertension, arrhythmias

Antivascular endothelial growth factor	Bevacizumab	Hypertension, thromboembolism

## Abbreviations

*α*-MHC: Alpha-myosin heavy chain
3D: Three-dimensional
ANP: Atrial natriuretic peptide
AP-1: Activator protein-1
APO-1: Apoptosis antigen-1
ASK-1: Apoptosis signal-regulating kinase 1
C3, C8, C9: Caspase-3, caspase-8, caspase-9
CARP: Cardiac adriamycin-responsive protein
CHF: Congestive heart failure
Cyto C: Cytochrome C
DISC: Death-inducing signaling complex
DOX: Doxorubicin
DOXol: Doxorubicinol
ECM: Extracellular matrix
FADD: Fas-associated death-domain
Fe(II): Ferrous
Fe(III): Ferric
eNOS: Endothelial nitric oxide synthase
FasL: Fas ligand
GPx: Glutathione peroxidase
GSH: Glutathione
H_2_O_2_: Hydrogen peroxide
iPS: Induced pluripotent stem
IREs: Iron-responsive elements
IRP-1: Iron regulatory proteins-1
MAPK: Mitogen activated protein kinases
MLC-2v: Ventricular myosin light chain-2
MMPs: Matrix metalloproteinases
NO: Nitric oxide
O_2_^∙−^: Superoxide anion
^∙^OH: Hydroxyl radical
ONOO^−^: Peroxynitrite
PARP: Poly(ADP-ribose) polymerase
PKC: Protein kinase C
PLCD: Phospholipase C delta
pro-MMPs: Precursors of matrix metalloproteinases
RIP: Receptor-interacting protein
RNS: Reactive nitrogen species
ROS: Reactive oxygen species
RYR2: Ryanodine receptor
SERCA2: Sarco-/endoplasmic reticulum Ca^2+^ ATPase
SOD: Superoxide dismutase
t-Bid: Truncated Bid
TNFR: TNF-*α* receptor
TRADD: TNFR-associated death domain protein
VSCC: Voltage-sensitive L-type calcium channel.
